# Leitlinienempfohlene standardisierte Instrumente bei multipler Sklerose: Eine inhaltlich vergleichende Analyse mittels der Internationalen Klassifikation der Funktionsfähigkeit, Behinderung und Gesundheit (ICF)

**DOI:** 10.1007/s00115-024-01752-z

**Published:** 2024-10-29

**Authors:** Jasmin Bolte, Elise-Marie Dilger, Anna Levke Brütt

**Affiliations:** 1 Department für Versorgungsforschung, Fakultät VI Medizin und Gesundheitswissenschaften, Carl von Ossietzky Universität Oldenburg, Ammerländer Heerstr. 114–118, 26129 Oldenburg, Deutschland; 2 Institut und Poliklinik für Medizinische Psychologie, Universitätsklinikum Hamburg-Eppendorf, Hamburg, Deutschland

**Keywords:** Encephalomyelitis disseminata, ICF, Neurologie, Rehabilitation, Funktionsfähigkeit, Encephalomyelitis disseminata, ICF, Neurology, Rehabilitation, Functional capacity

## Abstract

**Hintergrund:**

Leitlinien sind praxisorientierte Entscheidungshilfen und empfehlen die Verwendung standardisierter Instrumente zur Messung der Funktionsfähigkeit von Menschen mit multipler Sklerose (MS). Inwieweit diese durch die empfohlenen Instrumente erfasst wird, ist allerdings unklar.

**Ziel der Arbeit:**

In dieser Studie werden die Inhalte standardisierter Instrumente, die bei Menschen mit MS eingesetzt werden, mit der Internationalen Klassifikation der Funktionsfähigkeit, Behinderung und Gesundheit (ICF) abgeglichen, um die Verteilung der Inhalte auf die Funktionsdomänen und Kontextfaktoren zu beschreiben.

**Material und Methoden:**

Eingeschlossen wurden alle Instrumente, die in der S2k-Leitlinie „Diagnose und Therapie der Multiplen Sklerose, Neuromyelitis-optica-Spektrum-Erkrankungen und MOG-IgG-assoziierten Erkrankungen“ beschrieben wurden. Die Items der Instrumente wurden inhaltlich analysiert, indem sie in ihre sinnvollen Konzepte („meaningful concepts“ [MC]) eingeteilt und mittels standardisierter Regeln der ICF durch zwei Rater zugeordnet wurden.

**Ergebnisse:**

Die 23 eingeschlossenen Instrumente beinhalten 351 Items und 718 MC. Von diesen konnten 663 MC in der ICF abgebildet werden. 51 % (*n* = 340 MC) bezogen sich auf Körperfunktionen, 44 % (*n* = 291 MC) auf Aktivität und Teilhabe und 5 % (*n* = 32 MC) auf Umweltfaktoren. Das Kapitel Mobilität (d4) war mit *n* = 201 MC am stärksten in den Instrumenten vertreten. Die Interrater-Reliabilität lag bei k = 0,79.

**Diskussion:**

Die leitlinienempfohlenen Instrumente decken viele Bereiche der ICF ab, jedoch sind die Umweltfaktoren unterrepräsentiert. Die ICF-Verknüpfung erleichtert die Auswahl geeigneter Instrumente für Forschung und Praxis.

## Abstract (en)

**Background:**

Guidelines serve as practice-based decision support and recommend the use of standardized instruments to measure functional capacity in individuals with multiple sclerosis (MS). However, the extent to which a person’s functioning is captured by these instruments is unclear.

**Objectives:**

The aim of this study is to link the content of the MS disease guideline with the International Classification of Functioning, Disability and Health (ICF) in order to capture the distribution of the content on the functional domains and contextual factors and to uncover existing gaps.

**Materials and methods:**

Instruments of the S2k guideline “Diagnosis and therapy of multiple sclerosis, neuromyelitis optica spectrum diseases and MOG-IgG-associated diseases”, which were available free of charge in German or English and designed for adult people, were included. The instruments were classified into their meaningful concepts (meaningful concepts = MCs) and subsequently coded using standardized ICF rules by two raters.

**Results:**

The 23 included instruments contained 351 items and 718 MCs. Of these, 663 MCs could be linked to the ICF according to the coding rules. Of these, 51% (340 MCs) related to body functions, 44% (291 MCs) to the activity and participation component, and 5% (32 MCs) to environmental factors. The most frequently coded chapter was mobility (d4) with 201 MCs. Interrater agreement was k = 0.79.

**Discussion:**

The linking of the guideline-recommended instruments to the ICF shows that the instruments cover different themes. The area of environmental factors is underrepresented. The ICF linkage reveals a broad spectrum of functioning covered by the guideline-recommended instruments and facilitates the selection of appropriate instruments for the affected persons.

Multiple Sklerose (MS) ist die häufigste chronisch-entzündliche Erkrankung des zentralen Nervensystems junger Erwachsener [11]. Mithilfe unterschiedlicher Instrumente kann die Funktionsfähigkeit von an MS Erkrankten erfasst werden, der Verlauf der Erkrankung sowie Behandlungsergebnisse können dokumentiert werden [8]. In der S2k-Leitlinie der Deutschen Gesellschaft für Neurologie (DGN) werden verschiedene Instrumente zur Messung der Funktionsfähigkeit benannt, die eine Orientierung für die Forschung und Praxis bieten. Welche Bereiche der Funktionsfähigkeit diese Instrumente abdecken und ob eine Über- oder Unterrepräsentation von Bereichen der Funktionsfähigkeit vorliegt, wurde bisher nicht systematisch untersucht [8].

## Hintergrund und Fragestellung

Die MS, welche sich häufig im Alter von 20 bis 40 Jahren manifestiert, ist mit einer Prävalenz von 2,5 Mio. Fällen weltweit und einer Inzidenz von 8 bis 10 Fällen pro 100.000 Einwohner in Deutschland die häufigste entzündliche Erkrankung des Zentralnervensystems junger, erwachsener Menschen [9, 11, 13, 15]. Die Erkrankung führt zu einer progredienten fokalen Demyelinisierungen und zum Verlust von Nervenzellfasern. Die dadurch hervorgerufenen Symptome äußern sich durch neuropsychologische Symptome wie Fatigue, kognitive Einschränkungen und Depressionen, motorische Symptome wie Spastik und Einschränkungen der Mobilität und weitere Symptome wie Schmerzen, Sehstörungen, Störungen der Blasen- und Darmfunktion sowie sexuelle Dysfunktion [11].

Da die MS eine chronische Erkrankung ist, rücken die Auswirkungen der Erkrankung auf den Lebensalltag der Betroffenen in den Vordergrund. Um diese zu beschreiben, entwickelte die WHO im Jahr 2001 die Internationale Klassifikation der Funktionsfähigkeit, Behinderung und Gesundheit (ICF), welche auf dem biopsychosozialen Modell fußt [16]. Mit ihr kann systematisch mithilfe eines alphanumerischen Systems die Funktionsfähigkeit von Menschen beschrieben werden [8, 11].

Patient*innen berichten nicht immer spontan über ihre Beschwerden, wodurch ein regelmäßiges, standardisiertes Abfragen dessen nötig wird [6, 8]. Im Rahmen der deutschen MS-Leitlinie, welche von der Kommission „Leitlinie der Deutschen Gesellschaft für Neurologie“ herausgegeben wird, wird das aktuelle Wissen der MS zur Diagnostik und Therapie zusammengefasst und stellt somit einen Leitfaden für die behandelnden Berufsgruppen dar [8]. Die Leitlinie sollte möglichst alle Funktionsbereiche von an MS Erkrankten erfassen. Das Ausmaß, in dem die Instrumente der MS-Leitlinie die Funktionsfähigkeit und Kontextfaktoren berücksichtigen, ist jedoch unklar [7].

Ziel dieser Arbeit war es zu untersuchen, welche Domänen der ICF mit leitlinienempfohlenen Instrumenten erfasst werden können. Darauf aufbauend wurde herausgearbeitet, wie sich die Instrumente inhaltlich voneinander unterscheiden.

### Studiendesign und Untersuchungsmethoden

Auf Basis einer Literaturrecherche wurden im ersten Schritt Instrumente ausgewählt, die dann im zweiten Schritt inhaltlich mittels der ICF beschrieben wurden. Die erhobenen Datensätze können auf Anfrage von der korrespondierenden Autorin zur Verfügung gestellt werden.

### Auswahl von Instrumenten

Im ersten Schritt wurden alle in der 2021 überarbeiteten S2k-Leitlinie zur „Diagnose und Therapie der Multiplen Sklerose, Neuromyelitis-optica-Spektrum-Erkrankungen und MOG-IgG-assoziierten Erkrankungen“ [8] der Deutschen Gesellschaft für Neurologie (DGN) gelisteten Instrumente von zwei Personen unabhängig voneinander extrahiert und nach Abgleich zwischen diesen in der aktuellen Fassung beschafft. Eingeschlossen wurden Instrumente in deutscher oder englischer Sprache, die kostenlos zur Verfügung standen und für volljährige Menschen vorgesehen sind. Ausgeschlossen wurden somit Instrumente, die diese Kriterien nicht erfüllten, und solche, die keine Instrumente im eigentlichen Sinne darstellten, wie beispielsweise Kriterien zur weiterführenden apparativen Diagnostik oder auch Checklisten.

### ICF

Für den zweiten Schritt bildet die ICF die Arbeitsgrundlage. Die ICF wird in zwei Teile gegliedert. Der erste Teil befasst sich mit der Funktionsfähigkeit und Behinderung und stellt zum einen die Komponenten der Körperfunktionen (b) und der Körperstrukturen (s) und zum anderen die Aktivität und Partizipation (d) dar [8]. Als Körperfunktionen werden z. B. „mentale Funktionen“ oder „neuromuskuloskeletale und bewegungsbezogene Funktionen“ bezeichnet, unter Körperstrukturen werden „Strukturen des Nervensystems“ oder „mit der Bewegung in Zusammenhang stehende Strukturen“ gefasst. Eine Aktivität und Partizipation fokussieren auf „Kommunikation“ oder „Gemeinschafts-, soziales und staatsbürgerliches Leben“. Im zweiten Teil der ICF werden Kontextfaktoren, worunter die Umweltfaktoren (e), wie Unterstützung und Beziehungen und die personenbezogenen Faktoren, zählen, in der ICF erwähnt, jedoch aufgrund soziokultureller Unterschiede nicht klassifiziert wurden, beschrieben [8]. Jede der Komponenten besteht aus Domänen, welche wiederum in Kategorien als Einheiten der Klassifikation unterteilt werden [16]. Beispielsweise enthält die Komponente der Körperfunktionen die Domäne „mentale Funktionen“ (b1), welche weiter in Kategorien, wie zum Beispiel „Funktionen des Bewusstseins“ (b110), getrennt wird.

So bietet die ICF eine universelle Sprache zur Klassifikation der gesundheitsbezogenen Zustände und dient als Referenzinstrument zur Untersuchung der Inhalte von Datenquellen [16].

### Inhaltliche Analyse der Items mittels ICF

Für die anschließende Codierung auf Grundlage der ICF wurde ein systematisches und standardisiertes Vorgehen entsprechend den Regeln von Cieza et al. angewendet [3]. Die Items der im ersten Schritt eingeschlossenen leitlinienempfohlenen Instrumente wurden dafür in ihre sinnvollen Konzepte (= „meaningful concepts“ [MC]) getrennt. Nach dieser Vorgehensweise wird beispielsweise das Item „Während der letzten 14 Tage war ich aufgrund meiner Erschöpfung weniger aufmerksam“ des „Würzburger Erschöpfungsinventars bei MS“ (WEIMuS) (12) in die MC „Erschöpfung“ und „Aufmerksamkeit“ aufgeteilt. Die Trennung in MC erfolgte gemeinsam durch zwei Forscherinnen (JB und EMD). Anschließend wurden die MC von JB und EMD unabhängig voneinander anhand der Regeln nach Empfehlungen von Cieza et al. [3] mit der ICF-Klassifikation bis mindestens zur dreiziffrigen Ebene codiert. Zudem gab es die Möglichkeit, MC als nicht in der ICF definiert („not definable“ [nd]) oder als nicht durch die ICF abgedeckt („not covered“ [nc]) zu codieren. Die resultierende Codierung der beiden Rater wurde verglichen und bei mehrdeutigen Konzepten diskutiert, interpretiert und angepasst, bis Konsens gefunden wurde. Die Interrater-Reliabilität der dreiziffrigen Codierung wurde nach Abschluss der Codierung mittels Cohens Kappa berechnet.

## Ergebnisse

### Instrumente der MS-Leitlinie

Die 23 eingeschlossenen Instrumente erfüllten die Einschlusskriterien. Neben Instrumenten, die lediglich 1 Item umfassten, wurden auch längere Instrumente mit bis zu 88 Items eingeschlossen (Tab. 1).

**Tab. 1:** Eigenschaften der leitlinienempfohlenen Instrumente

Name	Items (*n*)	MC (*n*)	Leitliniengenannter Verwendungszweck [8]
*Visuelle Analogskala (VAS)*	1	1	Screening- und Rating-Tool zur Erfassung chronischer Schmerzen
*Subtest Alertness der Testbatterie zur Aufmerksamkeitsprüfung (TAP)*	1	1	Messung der Aufmerksamkeitsintensität im Rahmen einer neuropsychologischen Untersuchung zur Objektivierung der mentalen Fatigue
*Numerische Rating-Skala (NRS)*	1	1	Erfassung von tagesformabhängigen Schwankungen der spastischen Tonuserhöhung
*Bain Score for Tremor Severity*	1	6	Beurteilung des Tremor-Scores
*Ashworth-Skala*	6	8	Quantifizierung der Spastik
*Multiple Sclerosis Spasticity Scale (MSSS-88)*	88	92	Erfassung von tagesformabhängigen Schwankungen der spastischen Tonuserhöhung
*Berg Balance Scale (BBS)*	14	117	Neurologische Untersuchung der Beinfunktion auf Ataxie, Tremor und zur Einschätzung des Gleichgewichts
*Multiple Sclerosis Walking Scale-12 Test (MSWS-12-Test)*	12	18	Quantitative Beurteilung von Gangstörung und eingeschränkter Mobilität
*Timed „up and go“ (TUG)*	5	6	Beurteilung des Aufstehens
*Rivermead Mobility Index (RMI)*	15	21	Darstellung der signifikanten Besserung der nichtmedikamentösen Behandlung der Ataxie und des Tremors
*MS Functional Composite (MSFC)*			Messung von Behinderung durch MS
1. Timed 25-Foot Walk (T25FW)	1	1	Quantitativer Parameter zur Beurteilung einer Gangstörung und eingeschränkten Mobilität mittels einer standardisierten Gehstrecke von 7,62 m
2. Nine-Hole Peg Test (9-HPT)	1	2	Beurteilung von Ataxie und Tremor im Rahmen einer neurologischen Untersuchung für die Hand- und Armfunktion
3. Paced Auditory Serial Addition Test (PASAT)	1	4	Beurteilung von kognitiven Defiziten (nicht als isolierter Test empfohlen)
*Brief International Cognitive Assessment for MS (BICAMS)*			Basale und validierte Screeningverfahren für kognitive Defizite
1. Symbol Digit Modalities Test (SDMT)	1	4	„
2. California Verbal Learning Test (CVLT-II)	1	3	„
3. Brief Visual Memory Test Revised (BVMT-R)	1	2	„
*Würzburger Erschöpfungsinventar bei MS (WEIMuS)*	17	35	Standardisierter Fragebogen zur Quantifizierung der subjektiven Selbsteinschätzung der Fatigue
*Fatigue Skala für Motorik und Kognition (FSMC)*	20	42	Standardisierter Fragebogen zur Quantifizierung der subjektiven Selbsteinschätzung der Fatigue
*Hamilton-Depressionsskala (HDRS)*	21	41	Skala zur Beurteilung des Schweregrads einer Depression
*Multiple Sclerosis Impact Scale (MSIS-29)*	29	30	MS-spezifischer Fragebogen zur Erfassung von patient*innenberichteten Outcomes (= „patient-reported outcomes“ [PRO])
*Functional System Score (FSS)*	8	85	Beschreibung des primären Endpunkts bei der Schubbehandlung der MS
*Expanded Disability Status Scale (EDSS)*	20	41	Anwendung als klinischer Studienendpunkt und Messung des Grads der körperlichen Behinderung und der Gehfähigkeit
*Short Form 36 Health Survey Questionnaire (SF-36)*	36	52	Allgemeiner Fragebogen zur Erfassung von PRO
*Barthel-Index (BI)*	10	43	Strukturierte Feststellung der bestehenden Beeinträchtigungen und der Alltagsfähigkeiten
*Functional Independence Measure (FIM)*	18	24	Strukturierte Feststellung der bestehenden Beeinträchtigungen
*Brief Pain Inventory (BPI)*	9	21	Anamnese‑, Screening- und Rating-Tool zur Erfassung chronischer Schmerzen
*PainDETECT*	13	17	Anamnese‑, Screening- und Rating-Tool zur Erfassung chronischer Schmerzen
*Total*	351	718	–

### Verteilung der Codierungen über die ICF-Komponenten

Die eingeschlossenen Instrumente enthalten insgesamt 351 Items, von denen 718 „meaningful concepts“ (MC) abgeleitet wurden. Nach einer Probephase des Codierens durch JB und EMD zum Einüben der Codierregeln wurde die Übereinstimmung der beiden Codierenden berechnet, die eine gute Interrater-Variabilität ergab (Cohens Kappa: 0,79; [12]).

**Abb. 1:**
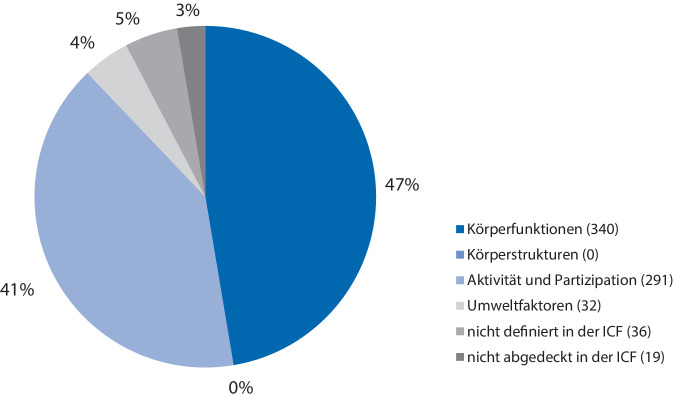
Verteilung der codierten sinnvollen Konzepte (*MC* „meaningful concepts“) auf den Komponenten der ICF

Mit den Komponenten der ICF konnten 663 MC verknüpft werden (Abb. 1). Entsprechend den Codierregeln wurden darüber hinaus 36 MC mit „nd“ („not definable“) und 19 MC mit „nc“ („not covered by ICF“) codiert [3].

Auf den Komponenten der ICF wurden die meisten Codierungen (*n* = 340, 47,4 %) dem Konstrukt der Körperfunktionen zugewiesen. Den Körperstrukturen hingegen wurden keine MC zugewiesen. Der Komponente der Aktivität und Teilhabe wurden 291 MC, also 41 % aller Konzepte zugewiesen. Bei den Kontextfaktoren wurden den Umweltfaktoren 32 MC und damit 4 % der MC zugeordnet. Die personenbezogenen Faktoren werden gegenwärtig nicht klassifiziert.

Die 663 mit der ICF verknüpften MC wurden mit 101 verschiedenen Kategorien der dreiziffrigen Ebene verknüpft. In der Komponente Körperfunktionen wurden 50 verschiedene Kategorien zugeordnet. Erkennbar ist dabei ein Verteilungsschwerpunkt auf dem Kapitel der mentalen Funktion (b1) mit 138 MC (40,6 %), in dem vor allem im Bereich der psychischen Energie und des Antriebs (b130) 43 MC sowie der emotionalen Funktionen (b152) 39 MC zugeordnet wurden.

Die Aktivität und Teilhabe präsentieren sich durch 45 verschiedene Kategorien, bei denen 201 MC und damit 69,1 % dieser Komponente mit dem Kapitel der Mobilität (d4) verknüpft wurden. Dies wurde vor allem durch das Instrument der „Berg Balance Scale“, bei welcher 92 MC diesem Kapitel zugeordnet wurden, verursacht.

Bei den Umweltfaktoren wurden die insgesamt 32 MC auf vier der fünf Kapitel innerhalb von sechs verschiedenen dreiziffrigen Codierungen verknüpft. Die Umweltfaktoren unterteilten wir ein weiteres Mal und überprüften, ob ein Umweltfaktor in einem beschriebenen MC als Bedingung für die Funktionsfähigkeit eines Menschen und somit als Teil des Tests gesehen wird oder ob ein Umweltfaktor ein Teil der Lebenswelt des alltäglichen Lebens des Menschen darstellt.

### Vergleich der Instrumente

**Abb. 2:**
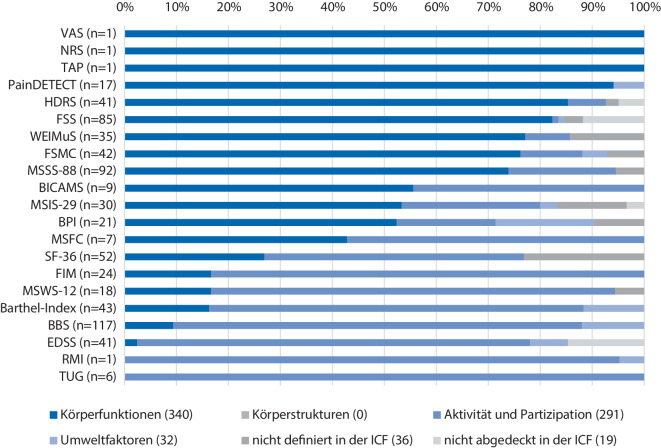
Verteilung der Instrumentencodierung mit den Komponenten der ICF (351 Items, 718 „meaningful concepts“)

Die leitlinienempfohlenen Instrumente variieren in ihrem Umfang, ihrem Inhalt, der Art der Erhebung und deren Zielen (Tab. 1). Fünf Instrumente erheben ausschließlich einzelne Domänen. So erfassen die Instrumente VAS, NRS, Subtest „Alertness“ der TAP und Bain Score for Tremor Severity mit jeweils nur einem Item und die Ashworth-Skala mit sechs Items ausschließlich Körperfunktionen (Abb. 2). Ein Fokus ausschließlich auf die Aktivität und Teilhabe besteht bei einem Instrument, dem TUG.

Eine kombinierte Erhebung von Körperfunktionen und der Aktivität und Partizipation erfolgt durch die Instrumente FIM, SF-36, HDRS, WEIMuS, BICAMS, MSFC, MSWS-12 und der MSSS-88. Schwerpunktmäßig wurde in der Komponente der Aktivität und Teilhabe das Kapitel Mobilität (d4) codiert. Bei BI und FIM ist ein Schwerpunkt der Codierung der Aktivität und Teilhabe im Bereich der Selbstversorgung (d5) zu erkennen.

Die Codierung der Umweltfaktoren fand lediglich bei neun Instrumenten statt, von denen drei Instrumente (MSIS-29, EDSS, BPI) die Lebenswelt der Betroffenen betrachteten und sechs Instrumente (RMI, FSS, FSMC, PainDETECT, Barthel-Index, BBS) als Bedingung für die Funktionsfähigkeit erhoben wurden. Kontextfaktoren werden ausschließlich in Kombination mit anderen Domänen erhoben. So kombiniert PainDETECT die Codierung der Körperfunktionen mit 16 MC und der Umweltfaktoren (b280 = Schmerzen) mit einem MC. Eine Kombination der Aktivität und Teilhabe mit den Umweltfaktoren erfolgt im RMI. Die drei Komponenten der Körperfunktionen, Aktivität und Teilhabe und der Umweltfaktoren wurden durch die Instrumente BPI, BI, EDSS, FSS, MSIS-29, FSMC und BBS codiert (Abb. 2). Dabei stellen die Umweltfaktoren jeweils den kleinsten Anteil dar. Die häufigste Codierung dieser Komponente fand mit 14 MC der BBS statt und wird der Bedingung für die Funktionsfähigkeit der Patient*innen zugeordnet.

## Diskussion

Ziel dieser Arbeit war es, leitlinienempfohlene Instrumente zu identifizieren und zu überprüfen, welche Domänen der ICF erfasst und inwieweit die verschiedenen Komponenten durch die Instrumente abgedeckt werden.

Die Ergebnisse zeigen, dass die leitlinienempfohlenen Instrumente sich in ihrem Umfang und dem Verwendungszweck unterscheiden. Die empfohlenen Instrumente unterscheiden sich in ihrer Länge. Zu berücksichtigen ist, dass die Anzahl der MC der Instrumente sich durchaus unterscheidet und beispielsweise die BBS mit *n* = 117 MC auf 14 Items die meisten MC besitzt.

Zudem decken sie unterschiedliche Aspekte der ICF ab. Die Körperfunktionen und insbesondere mentale Funktionen werden durch WEIMuS, FSMC, HDRS und MSIS-29 abgedeckt.

Die Funktionen des urogenitalen und reproduktiven Systems wurden vorwiegend durch sieben MC des FSS codiert. Den Funktionen der Haut und der Hautanhangsgebilde wurde hingegen kein MC der Instrumente zur MS-Erkrankung zugeordnet.

Die Körperstrukturen wurden nicht codiert, was durch den initialen Ausschluss der Tests mit apparativen diagnostischen Kriterien und Checklisten erklärbar ist.

Da Symptome wie Blasen‑, Darm- und Sexualfunktionsstörungen unter anderem für die berufliche Leistungsfähigkeit von Bedeutung sind, jedoch häufig nicht von Betroffenen berichtet werden, ist es erforderlich, diese gezielt beispielsweise mittels Checklisten zu erfragen [6, 8, 9, 11]. Solche Checklisten wurden in der Leitlinie aufgeführt, um im Rahmen der Anamnese eingesetzt zu werden. Empfohlene Instrumente gingen jedoch nur selbst auf diese Funktionsstörungen ein. Eine systematische Verlaufsbeobachtung und eine Einbettung in ein biopsychosoziales Krankheitsverständnis können jedoch nur erschwert erfolgen. Die neuropsychologischen Aspekte hingegen wurden ausführlich innerhalb der Instrumente betrachtet, was im Einklang mit deren Bedeutsamkeit für die Lebensqualität der Erkrankten ist [11].

Bei der Codierung der Komponente Aktivität und Teilhabe liegt ein klarer Schwerpunkt auf der Mobilität. Ein großer Anteil dieser Codierung ist dabei lediglich einem Instrument, der Berg Balance Scale (BBS), zuzuordnen (Abb. 2). Jedoch ist zu beachten, dass die Items der BBS mehrere unterschiedlich beschriebene Bereiche umfassen und somit beispielsweise das fünfte Item sechs Mal mit dem Code d4200 (sich beim Sitzen verlagern) codiert wurde. Dies könnte bei der BBS zu einer Überschätzung der Tiefe führen.

Allen weiteren Kategorien der Komponente der Aktivität und Partizipation wurden jeweils mehrere MC zugeordnet, wodurch die Bedeutung der sozialen und gesellschaftlichen Perspektive der Funktionsfähigkeit des Menschen in der Leitlinie aufgedeckt wird.

Im Vergleich dazu waren die Umweltfaktoren, die mit lediglich *n* = 32 MC innerhalb von nur neun Instrumenten codiert wurden, unterrepräsentiert. Bei 14 Instrumenten wurden somit keine Umweltfaktoren codiert. Zudem fand keine Codierung im Bereich der Einstellungen (e4) statt, wodurch lediglich vier der fünf Kategorien verknüpft wurden. Die Einstellungen beeinflussen auf allen Ebenen das soziale und individuelle Verhalten [16]. Dadurch werden Konzepte wie beispielsweise Stigmatisierung nicht erfasst.

Die Unterpräsentation der erfassten Umweltfaktoren ist auch in anderen Studien zu beobachten [1]. Die Berücksichtigung der Kontextfaktoren ist allerdings für die Betroffenen, insbesondere für deren medizinische Versorgung, relevant [14]. So berichten an MS Erkrankte beispielsweise, dass die Einstellung anderer Personen sowie auf die Bedürfnisse angepasste Hilfsmittel wichtig für die Lebensqualität sind [4]. Jedoch sind für die Erfassung kaum Erhebungs- oder Dokumentationsinstrumente vorhanden [1]. Neben BPI und BBS umfasst auch der BI die Komponenten Körperfunktionen, Aktivitäten und Teilhabe und Umweltfaktoren mit jeweils drei oder mehr MC. Dabei sollte nach der ICF die Betrachtung der Funktionsfähigkeit ganzheitlich gesehen werden, wodurch auch Kenntnisse der Kontextfaktoren, die mit den weiteren Komponenten der ICF interagieren und sich beeinflussen, erforderlich sind [1, 2].

Insgesamt zeigt sich die ICF als ein nützliches Mittel zur standardisierten Übersetzung der Inhalte von Datenquellen, in diesem Fall standardisierter Instrumente, womit Inhalte verglichen und Lücken aufgedeckt werden können. Dadurch kann die Auswahl eines für den Betroffenen geeigneten Instruments und der zu erhebenden Eigenschaften vereinfacht und eine sinnhafte Kombination für die Erhebung der Funktionsfähigkeit erwogen werden [6]. Übersichtsarbeiten zeigen auf, dass es neben den Instrumenten, die in der Leitlinie aufgeführt werden, diverse andere fundierte Instrumente zur Erfassung der Funktionsfähigkeit von an MS Erkrankten gibt [5, 10]. Im klinischen Alltag stehen also verschiedene Instrumente, die nicht in der Leitlinie genannt werden, zur Verfügung und können zur Erfassung der Funktionsfähigkeit eingesetzt werden. Zukünftige Studien können vertieft zum Forschungsgegenstand nehmen, wie sich die tatsächliche Nutzung der leitlinienempfohlenen Instrumente in der Praxis darstellt und wie die weiteren Instrumente, die nicht in der Leitlinie aufgeführt werden, genutzt werden.

### Limitationen

Die Arbeit bezieht sich auf die S2k-Leitlinie der DGN aus dem Jahr 2021. Eine Aktualisierung fand am 31.11.2023 statt. Instrumente, die in der Leitlinie erhalten bleiben, können durch diese Studie jedoch weiterhin hinsichtlich ihrer inhaltlichen Strukturierung eingeordnet und bewertet werden. Auch kann die Studie für zukünftige Anpassungen der Leitlinie eine Orientierung bieten.

Umweltfaktoren waren häufig sehr unspezifisch angegeben und wurden als Zusatzinformation codiert. Dieses geringe Maß an Spezifität könnte zu der Unterrepräsentation beigetragen haben.

Um die Subjektivität der Codierung zu reduzieren, erfolgte die Codierung unabhängig durch zwei Forschende. Mit diesem Verfahren konnte eine gute Intercoder-Reliabilität erreicht werden. Als Limitation ist aufzuführen, dass nicht mehr Personen bei der unabhängigen Codierung beteiligt waren und im Falle mehrdeutiger Konzepte eine Konsensbildung erfolgte, keine strukturierte Konsensfindung mittels systematischer Verfahren.

## Fazit für die Praxis

- Die insgesamt 23 leitlinienempfohlenen Instrumente unterscheiden sich in ihrem Umfang und dem Verwendungszweck und erfassten am häufigsten die Körperfunktionen, gefolgt von der Aktivität und Partizipation. Dennoch wird zum Beispiel die Blasenfunktion nur durch zwei der eingeschlossenen Instrumente mit mehr als einem Item erfasst.
- Die Umweltfaktoren sind, trotz ihrer Relevanz für die Betroffenen, gänzlich unterrepräsentiert und sollten für eine reale Versorgungssituation der Betroffenen stärker einbezogen werden.
- Die Ergebnisse dieser Arbeit bieten eine Orientierung und auch eine Anleitung, welche Instrumente für welche Bereiche der Funktionsfähigkeit des Betroffenen im Rahmen einer interdisziplinären medizinischen Versorgung erhoben und kombiniert werden können.
- Um biopsychosoziale Aspekte der MS zu erfassen, ist eine Kombination verschiedener Instrumente sinnvoll. Darüber hinaus können auf Basis der Codierung Instrumente ausgewählt werden, um Krankheits- und Therapieverläufe zu beobachten und zu evaluieren.
- Für die praxisnahe Versorgung sind einige der hier aufgeführten Instrumente, abseits eines Forschungssettings, zu aufwendig. Daher sind eine bewusste Instrumentenauswahl anhand der Funktionsfähigkeit sowie der interdisziplinäre Austausch wichtig.

## References

[1] Bülau NI, Kessemeier F, Petermann F et al (2016) Evaluation von Kontextfaktoren in der psychosomatischen Rehabilitation. Rehabilitation 55(6):381–387. 10.1055/s-0042-119897 27923243 10.1055/s-0042-119897

[2] Büttner C, Quindel R (2013) ICF als bio-psycho-soziales Modell von Gesundheit. In: Gesprächsführung und Beratung. Springer, Berlin, Heidelberg, S. 75–88 10.1007/978-3-642-30212-1-5

[3] Cieza A, Brockow T, Ewert T et al (2002) Linking health-status measurements to the international classification of functioning, disability and health. J Rehabil Med 34(5):205–210. 10.1080/165019702760279189 12392234 10.1080/165019702760279189

[4] Deutsche Vereinigung für Rehabilitation (2007) Für eine optimierte Versorgung mit Hilfsmitteln. https://www.dvfr.de/fileadmin/user_upload/dvfr/downloads/stellungnahmen/dvfr-hilfsmittel-expertise_061017.pdf. Zugegriffen: 30. Apr. 2024

[5] Ezegbe C, Zarghami A, van der Mei I et al (2023) Instruments measuring change in cognitive function in multiple sclerosis: A systematic review. Brain Behav 13(6):e3009. 10.1002/brb3.3009 37062948 10.1002/brb3.3009 PMC10275522

[6] Flachenecker P, Dettmers C, Henze T (2019) Rehabilitation bei Multipler Sklerose: multimodal, interdisziplinär, wirksam. Neuro U2d 2(02):171–187. 10.1055/a-0803-5493

[7] Guo J, Cheng C, Yan W et al (2014) Systematic review of clinical practice guidelines related to multiple sclerosis. Plos One 9(10):e106762. 10.1371/journal.pone.0106762 25302678 10.1371/journal.pone.0106762 PMC4193735

[8] Hemmer B et al (2021) Diagnose und Therapie der Multiplen Sklerose, Neuromyelitis-optica-Spektrum Erkrankungen und MOG-IgG-assoziierten Erkrankungen, S2k-Leitlinie. www.dgn.org/leitlinien. Zugegriffen: 30. Apr. 2024

[9] Holstiege J, Steffen A, Goffrier B et al (2017) Epidemiologie der Multiplen Sklerose – Eine populationsbasierte deutschlandweite Studie. Zentralinstitut für die kassenärztliche Versorgung in Deutschland 10.20364/VA-17.09

[10] Kamińska J, Koper OM, Piechal K et al (2017) Multiple sclerosis—etiology and diagnostic potential. Postepy Hig Med Dosw 71(0):551–563. 10.5604/01.3001.0010.3836 10.5604/01.3001.0010.3836 28665284

[11] Kip M, Schönfelder T, Bleß H‑H (2016) Hrsg. Weißbuch Multiple Sklerose, 1. Aufl. Springer, Berlin, Heidelberg 10.1007/978-3-662-49204-8

[12] Kuckartz U, Rädiker S (2022) Qualitative Inhaltsanalyse. Methoden, Praxis, Computerunterstützung, 5. Aufl. Beltz Juventa, Weinheim, Basel

[13] MS Forschungs- und Projektentwicklungs-gGmbH (18.01.2022) Das MS-Register. https://www.msregister.de/ms-register/das-ms-register/. Zugegriffen: 18. Jan. 2022

[14] Stumm J (2021) Koordination der Versorgung multimorbider Patient*innen. Konzepte zur Unterstützung der Hausärzt*innen. Charité – Universitätsmedizin, Berlin 10.17169/REFUBIUM-29133

[15] Weih M, Roßnagel F, Dikow H et al (2020) Daten zur Multiplen Sklerose in Deutschland und ihre Abbildung im Register des ambulanten Netzwerkes NeuroTransData (NTD). Fortschr Neurol Psychiatr 88(6):379–385. 10.1055/a-1130-6222 32557466 10.1055/a-1130-6222

[16] WHO (Hrsg) Internationale Klassifikation der Funktionsfähigkeit, Behinderung und Gesundheit (Stand Oktober 2005). Genf: WHO. Bundesinstitut für Arzneimittel und Medizinprodukte, Köln

